# Direct torque control for a six phase induction motor using a fuzzy based and sliding mode controller

**DOI:** 10.1038/s41598-025-97479-1

**Published:** 2025-04-28

**Authors:** Mohamed I. Abdelwanis, Alaa A. Zaky, F. Selim

**Affiliations:** Electrical Engineering Department, Faculty of Engineering, Kafrelsheikh University, Kafr El-Sheikh, Egypt

**Keywords:** Modified six-phase induction motor, SVPWM, Fuzzy-PID, DTC, SMC, Engineering, Mathematics and computing

## Abstract

Direct Torque Control (DTC) is widely recognized for its fast dynamic response and simplicity in controlling induction motors. However, conventional DTC suffers from drawbacks such as high torque and flux ripple, sensitivity to parameter variations, and poor performance under low-speed operation. This study proposes an enhanced DTC strategy for a modified six-phase induction motor (MSPIM) by integrating Fuzzy-Based Proportional-Integral-Derivative (FPID) control compared with conventional PID and Sliding Mode Control (SMC). The FPID, PID, and SMC controller are employed to regulate speed and flux, leveraging its adaptability and robustness to system uncertainties. The six-phase induction motor, with its inherent fault-tolerant capabilities and reduced torque pulsations, serves as an ideal candidate for high-performance applications. Results obtained using MATLAB Simulink show that the proposed control strategy significantly reduces torque and flux ripple, improves dynamic response, and enhances robustness against speed and load changing. This work highlights the potential of combining intelligent control techniques like FPID, PID and SMC to advance the performance of DTC in multi-phase induction motor drives, particularly in applications requiring high reliability and efficiency. Based on simulation results, the fuzzy PID inverter reduces the speed error and THD of the current and voltage waveforms, thereby improving MSPIM’s overall performance when compared to the regular PID and SMC.

## Introduction

Direct Torque Control (DTC) is a widely adopted technique for controlling multi-phase induction motors, renowned for its rapid torque and flux response capabilities. However, conventional DTC methods often encounter challenges such as significant torque and flux ripples, sensitivity to parameter variations, and suboptimal performance at low speeds. To mitigate these issues, various control strategies have been explored, including Proportional-Integral-Derivative (PID) controllers, and Sliding Mode Control (SMC). Each of these approaches offers distinct advantages and limitations.

This study introduces a Fuzzy-PID controller as an advanced approach to enhance DTC performance in six-phase induction motors. By integrating fuzzy logic with traditional PID control, the Fuzzy-PID controller aims to combine the adaptability and robustness of fuzzy systems with the precision and simplicity of PID controllers, thereby improving overall system performance.

### Background and significance

Six-Phase Induction Motors (SPIM): These motors are gaining prominence in applications requiring high reliability and power density, such as electric vehicles and aerospace systems^1^. Their multi-phase configuration offers benefits like reduced torque pulsations and enhanced fault tolerance. However, the complexity of SPIMs necessitates sophisticated control strategies to fully leverage these advantages.​

Direct Torque Control (DTC): DTC provides a simplified control structure with rapid dynamic response^2^. Despite its benefits, traditional DTC suffers from drawbacks including high torque and flux ripples and sensitivity to motor parameter variations, which can adversely affect performance^3^.

PID Controllers: Known for their straightforward implementation and effectiveness in various applications, PID controllers can struggle with tuning challenges and may not adequately address the nonlinearities inherent in SPIMs^4^.

Sliding Mode Control (SMC): SMC is recognized for its robustness against system uncertainties and external disturbances. However, it is often associated with chattering effects, which can lead to mechanical wear and reduced system lifespan^5^.

Fuzzy-PID Controllers: By integrating fuzzy logic with traditional PID control, Fuzzy-PID controllers aim to enhance system adaptability and robustness^6^. This hybrid approach allows for dynamic adjustment of PID parameters based on system behavior, potentially leading to improved performance in terms of stability, transient response, and resilience to disturbances.

### Literature review

A six-phase motor was intended to solve the low-order torque ripple issue caused by a three-phase converter operating in six stages^7^. According to^3^, multiphase machines with a phase number higher than five have various features over their three-phase equivalents, such as smaller electrical torque distortion, better fault tolerance, more power density, etc.. This is because of these features and the rising requirements placed on the multi-phase of IM. When one or more phases are open circuits in^8^, the MMF of the stator is maintained constant to create uniform torque. A multiphase IM’s dynamic and steady-state behavior under fault conditions were examined in^9^. In^10^, a new structure based on the use of three sensors of current is presented for 6-phase IM using vector control. Reference^11^ develops an analysis of performance for the MSPIM.

Direct Torque Control (DTC) is a widely used control strategy for electric motors due to its fast dynamic response, simplicity, and absence of complex coordinate transformations^2^. Conventional DTC suffers from high torque and flux ripple due to the use of hysteresis controllers and limited voltage vectors, which degrade motor performance, The switching frequency is not constant, leading to increased harmonic distortion and acoustic noise^12^, Conventional DTC struggles to maintain precise control at low speeds, resulting in poor torque regulation and increased losses, and the performance of conventional DTC is highly sensitive to variations in motor parameters, such as stator resistance and inductance^13^. To implement the DTC concept, the fundamentals of electrical torque and magnetic flux control, as well as the truth table of switching, are first taught^14^. The driving behavior is then illustrated. along with the switching techniques and the impact of the electrical torque and magnetic flux hysteresis band magnitude. The link between the supply voltage and the accompanying torque change over the course of a cycle has received special attention^15^. DTC of Dual Star Induction Motor by using Grey Wolf Optimization algorithm^16^, and for modified DTC by FLC of double fied induction motor^17^. A GA-based MRAS controller used to enhance DTC for sensorless IM^18^. Neuro-fuzzy control of an IM, Neuro-fuzzy control is used to improve DTC of an IM^19^. A space vector modulated DTC-connected solar water pumping system^20^, A battery-electric vehicle’s whole traction motor control system uses DTC-SVM^21^.

The SVPWM, sinusoidal PWM (SPWM), offset injection, and harmonic injection methods are extensively investigated to get the best three-phase inverter output voltage^22^. The SPWM inverters offer greater flexibility and ease of use. However, the output voltage waveforms have higher harmonic components than usual, which lowers the efficiency^23^. A greater phase number increases the complexity of the SVPWM inverter. For a multi-phase inverter, a switching mechanism is required to be better and simpler to get around the complexity that comes with having more phases^24^.

An effective instrument that can improve the performance of the electrical machinery in power systems is fuzzy logic^25^. The development of sliding mode and fuzzy logic controllers to improve operation and achieve high-performance placement of six-phase IM is described in^26,27^.To improve overall system performance as determined by a cost function, the PID controller’s parameters were programmed using fuzzy logic tuners^28,29^. Using fuzzy-based-PI control of the PMSM to control the motor speed in the hybrid power method^30^, there is a large literature that provides an evaluation of the PID control strategy along with the backstepping control strategy in different cases, including the proposed flight mission in obstacle-free surroundings and obstacle-filled environments, as in^31^. Fuzzy PID controller on DTC of dual star induction motor tuned by particle swarm optimization and genetic algorithm^32^.

Sliding Mode Control (SMC) is a robust control technique that is highly effective in dealing with system uncertainties and disturbances^33^. SMC has been integrated with DTC to improve its performance under parameter variations and load disturbances of induction motor^34^. The combination of SMC and DTC has been shown to reduce torque and flux ripple while maintaining fast dynamic response^35^.

### Motivation

When industrial high-power applications are needed, one of the most palatable solutions is six-phase IM. Six-phase IM has recently become more popular in high-rating uses in place of six-phase synchronous motors due to the latter’s lighter weight at the same power^36^. Additionally, compared to three-phase IM, the double three-phase IM produces more output torque. The IM is excellent for higher ratings and/or large current requirements, such as electric aerospace applications, ship drive, and electric vehicles, due to its higher operational torque characteristic^37^.

At compared the standard three-phase IM utilized in industrial applications, with dual three-phase IM integration has numerous advantages^4^. Enhanced stability, lowered harmonic flux, fewer electrical torque pulsations, and a smaller static power converter are some of these benefits. Apart from these benefits, another characteristic of multi-phase injection molding applications compared to three-phase IM is their ability to function even if one or more stator phases lose excitation^38^.

The objective of this study is to enhance the performance of MSPIMs that drive variable mechanical loads. In recent years, six-phase induction motors have gained significant attention for their advantages over traditional three-phase motors, including reduced torque ripple, lower harmonic content, and improved fault tolerance. However, the implementation of DTC for six-phase induction motors presents unique challenges and opportunities, necessitating a thorough analysis of conventional methods and their limitations. The study presents the modeling and analysis of conventional PID, SMC and ideal FPID controllers for MSPIM. The proposed control strategy is shown to have advantages over traditional PID and SMC controllers, particularly in transient control and offering fast and accurate speed tracking, as demonstrated by comprehensive simulation results.

The article is divided into several sections. Section “System description and mathematical model of MSPIM” presents an MSPIM mathematical model. Section "Six-phase VSI modulation" presents the SVPWM six-phase inverter modeling. Section "Model of DTC-SVPWM inverter" analyzes the DTC modeling for the 6-phase inverter. Section "The proposed FPID controller and SMC of MSPIM" describes a proposed fuzzy-PID and SMC controller for SPIM. In Section "Applications", the application of the paper is discussed. Finally, the output discussion and conclusions of the paper are concluded in Section "Conclusions".

### Identified research gaps

1. Lack of comparative studies with modern control methods

Issue: Most FPID-DTC studies compare only with classical PID or basic DTC, ignoring advanced methods like, Sliding Mode Control (SMC) (robust but chattering-prone).

Consequence: The relative merits of FPID vs. these methods remain unclear.

2. Inadequate handling of multi-phase asymmetry

Issue: Six-phase motors introduce asymmetric winding faults or parameter imbalances, but FPID-DTC studies assume ideal symmetry.

Consequence: Performance degrades in real-world fault conditions (e.g., open-phase scenarios).

3. No standardized FPID tuning methodology

Issue: Existing FPID designs rely on, Heuristic rule bases (non-optimal for all operating points), Fixed membership functions (lack adaptability under dynamic loads).

Consequence: Suboptimal torque ripple suppression and efficiency losses.

4. Limited real-time validation

Issue: Most FPID-DTC studies are simulation-only (MATLAB/Simulink) or use small-scale lab prototypes.

Consequence: Unproven scalability for industrial high-power applications .

### Paper innovations

This article’s primary contribution can be summed up as follows:

- To improve torque pulsation and motor reliability, a 3-phase IM is converted to a 6-phase IM.
- This study models and analyzes fuzzy-based PID, SMC and PID controllers used to drive an MSPIM system that operates at varied speeds.
- An FPID controller was suggested to maintain the MSPIM mechanical speed at the defined reference speed connected to the operating condition.
- Direct torque control SVPWM closed-loop inverter MSPIM speed control is introduced, and the operation results are examined.

## System description and mathematical model of MSPIM

**Fig. 1:**
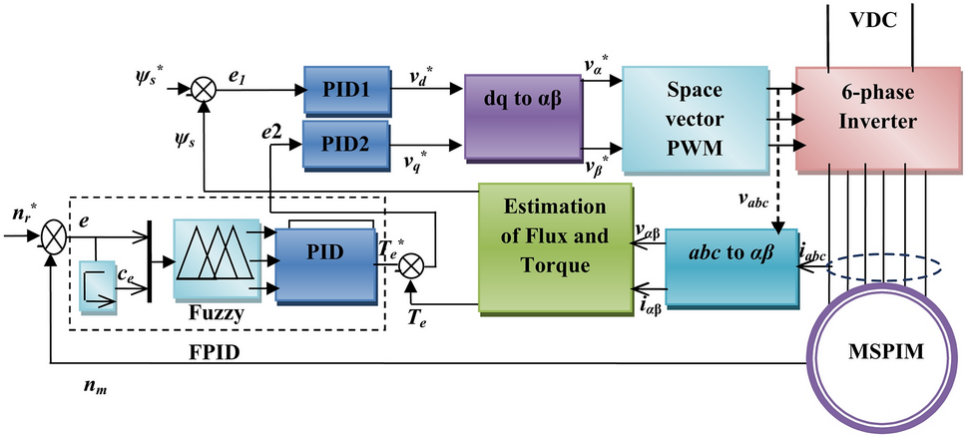
The proposed fuzzy-PID closed loop Schematic diagram of MSPIM.

The fuzzy-based PID controller block diagram used to operate a DTC MSPIM drive is shown in Fig. 1. The rotational speed fuzzy-based PID controller, flux PID controller, and torque PID controller comprise the main DTC system components, the observer of the torque and the stator flux, etc.

The six-phase induction motor can be modeled using voltage, flux, and torque equations in the the synchronous reference frame simplifies the analysis and control design. The transformation is applied to both stator and rotor quantities. Some assumptions are used in order to build the d-q MSPIM model, and they are as follows: uniform air gap, sinusoidally distributed windings. The core does not have magnetic saturation or core loss.

The waveforms of six phase voltages for MSPIM are written as:

1 $$\left\{ {\begin{array}{*{20}c} {v_{as} = V_{m} \sin (\omega t)} \\ {v_{bs} = V_{m} \sin (\omega t - \pi /3)} \\ {v_{cs} = V_{m} \sin (\omega t - 2\pi /3)} \\ {v_{ds} = V_{m} \sin (\omega t - \pi )} \\ {v_{es} = V_{m} \sin (\omega t - 4\pi /3)} \\ {v_{fs} = V_{m} \sin (\omega t - 5\pi /3)} \\ \end{array} } \right.$$

The d-q axis stator voltages of MSPIM are written as^39^:

2 $$v_{q} = \frac{2}{6}\left( {\mathop \sum \limits_{k = 1}^{6} v_{k} \cos (\theta - \left( {k - 1} \right)\pi /6)} \right)$$

3 $$v_{d} = \frac{2}{6}\left( {\mathop \sum \limits_{k = 1}^{6} v_{k} \sin (\theta - \left( {k - 1} \right)\pi /6)} \right)$$

The quadrature-axis flux and current stator and rotor of MSPIM are written as:

4 $$\psi_{qs} = 1/s\left( {v_{qs} - R_{s} i_{qs} - \omega_{e} \psi_{ds} } \right)$$

5 $$i_{qs} = 1/L_{s} \left( {\psi_{qs} - L_{m} i_{qr} } \right)$$

6 $$\psi_{qr} = 1/s\left( { - R_{r} i_{qr} + \psi_{dr} \left( {\omega_{r} - \omega_{e} } \right)} \right)$$

7 $$i_{qs} = 1/L_{r} \left( {\psi_{qr} - L_{m} i_{qs} } \right)$$

Direct-axis flux and current can stator and rotor of MSPIM be written as:

8 $$\psi_{ds} = 1/s\left( {v_{ds} - R_{s} i_{ds} + \omega_{e} \psi_{qs} } \right)$$

9 $$i_{ds} = 1/L_{s} \left( {\psi_{ds} - L_{m} i_{dr} } \right)$$

10 $$\psi_{dr} = 1/s\left( { - R_{r} i_{dr} + \psi_{qr} \left( {\omega_{r} - \omega_{e} } \right)} \right)$$

11 $$i_{dr} = 1/L_{r} \left( {\psi_{qr} - L_{m} i_{ds} } \right)$$

The electrical torque and mechanical speed of MSPIM are:

12 $$T_{d} = 3p/2\left( {\psi_{ds} i_{qs} - \psi_{qs} i_{ds} } \right)$$

13 $$\omega_{r} = p/2s\left( {1/j\left( {T_{d} - T_{L} - 2D\omega_{r} /p} \right)} \right)$$

The motor-mechanical balance equation is wrtien as:

14 $$T_{d} = T_{L} + J\frac{{d\omega_{m} }}{dt} + D\omega_{m}$$

## Six-phase VSI modulation

**Fig. 2:**
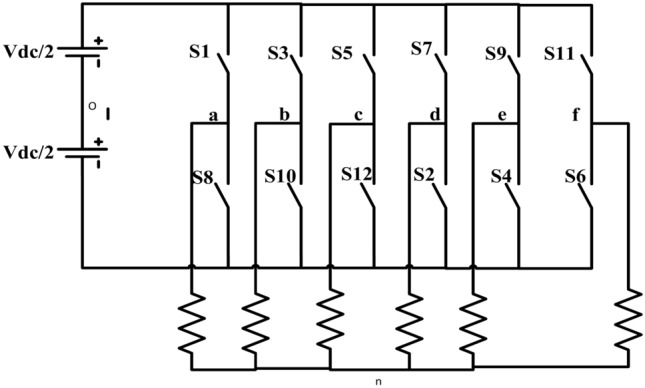
The power circuit of the six-phase inverter.

Figure 2 depicts the circuit configuration of a six-phase voltage source inverter SPVSI powering MSPIM^40^. Six-phase induction motor output phases are designated as a, b, c, and d, e, f, and inverter leg voltage as A, B, C, D, E, and F. Inverter leg voltages can be used to realize phase voltages in the following ways^41^:

15 $$\left[ {\begin{array}{*{20}c} {v_{an} } \\ {v_{bn} } \\ {v_{cn} } \\ {v_{dn} } \\ {v_{en} } \\ {v_{fn} } \\ \end{array} } \right] = \frac{2}{3}\left[ {\begin{array}{*{20}c} {v_{A} } \\ {v_{B} } \\ {v_{C} } \\ {v_{D} } \\ {v_{E} } \\ {v_{F} } \\ \end{array} } \right] - \frac{1}{3}\left[ {\begin{array}{*{20}c} {v_{B} + v_{C} } \\ {v_{C} + v_{A} } \\ {v_{A} + v_{B} } \\ {v_{E} + v_{F} } \\ {v_{F} + v_{D} } \\ {v_{D} + v_{E} } \\ \end{array} } \right]$$

The space vector for fixed-frame MSPIM using power-invariant transforms can be defined as^40^:

16 $$v_{dq} = v_{q} + j v_{d} = \frac{2}{6}\left( {v_{a} + a^{16} v_{b} + a^{8} v_{c} + av_{d} + a^{5} v_{e} + a^{9} v_{f} } \right)$$

where, *a* = *exp*(*j*π/6).

**Fig. 3:**
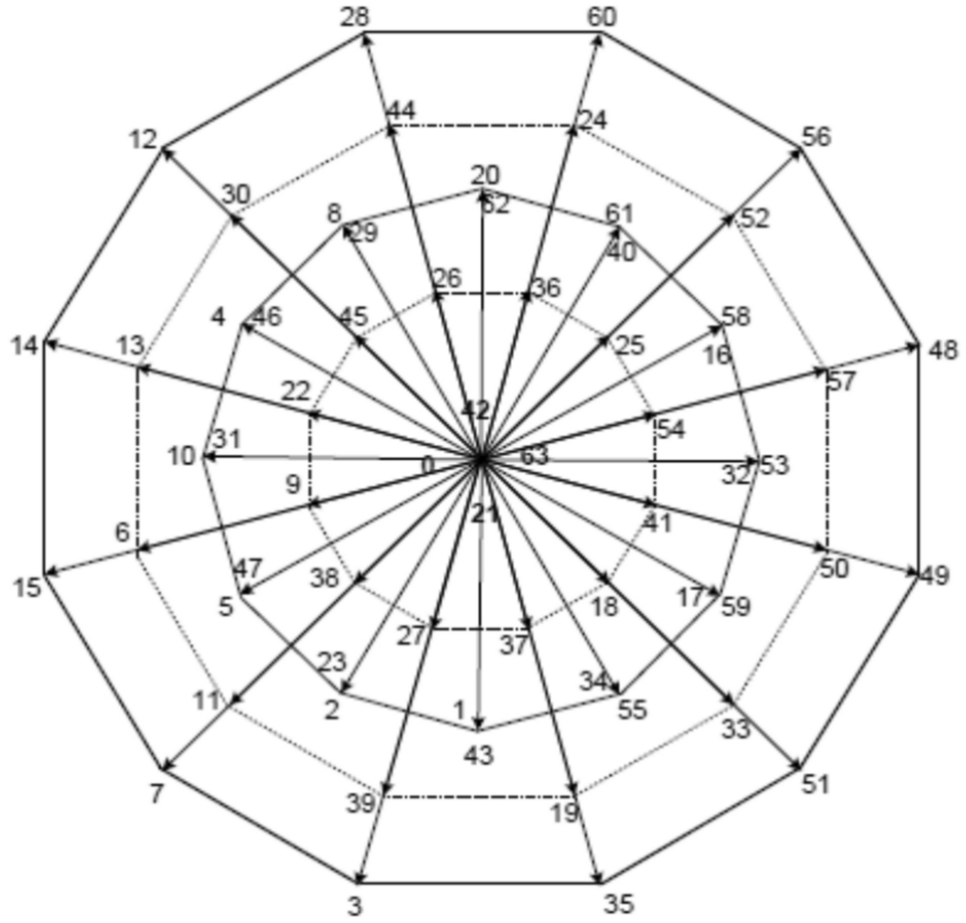
Vectors of the SPVSI phase voltage in the d-q plane.

Figure 3 shows the space vectors of SPVSI on the d-q axis. This contains 60 active vectors and 4 vectors empty that create four 12-sided polygons in a circle. The third-biggest vectors may be created by any concerning the two switching state mixtures since 12 of the 60 vectors active are frequent and lie along the third-biggest vectors. Zero vectors are transferred to the origin. These d-q axis variables are in charge of the rotating MMF and generate electromechanical energy. Therefore, in general, SVPWM techniques only use the big vectors in the d-q axis to reduce stator losses. In Fig. 3, the classification of vectors can be seen. Certain criteria must be met by a space vector modulation technique, such as maintaining a fixed switching frequency and avoiding unnecessary switching to reduce switching losses. It must be possible to use all of the DC bus sides that are available. To achieve a high AC voltage, lower harmonic contents in the AC voltage must be minimized.

The vector space division approach requires complicated implementation processes because the frames of switching are transferred to the inverter instead of the frames of the machine. A straightforward SVPWM known as a technique for classifying vectors was proposed in^40^, where an SPVSI is treated as two three-phase inverters for operational purposes.

The pulses for an SP VSI are generated using two identical conventional SVPWM processes in this vector method, with the second SVPWM’s phase vector reference being shifted by 30° electrical. Switching time calculations can be made using the three-phase SVPWM formula:

17 $$T_{1} = \frac{2}{\sqrt 3 }m_{a} T_{s} \sin \left( {60 - \theta } \right)$$

18 $$T_{2} = \frac{2}{\sqrt 3 }m_{a} T_{s} \sin \left( \theta \right)$$

19 $$T_{0} = T_{s} - T_{1} - T_{2}$$

Specifically, ma = v*vl $m_{a} = \left| {v^{*} } \right|/\left| {v_{l} } \right|$ Where |*v*_*l*_| is the length of the greatest vector and |v^*^| is the reference space vector. The greatest m_a_ value that used to be attained is 0.89655 and a 0.5977 VDC equivalent phase voltage^40^ when the phase shift of the reference space vector is 30 degrees electrical. As a result, certain vectors will be inserted right away.

## Model of DTC-SVPWM inverter

Figure 1 depicts the structure of the DTC system^42^. Model U-II can be used to determine how to manage the flux of the stator in direct torque control systems^43^. The relationship between the stator voltage and flux of the stator is shown below. In low-speed operation, the control system uses a sub-circular flux path, which ensures constant electrical torque and stator flux at a small frequency. The DTC voltage formulas are^44^.

20 $$v_{s\alpha } = R_{s} i_{s\alpha } + \psi_{s\alpha }$$

21 $$v_{s\beta } = R_{s} i_{s\beta } + p\psi_{s\beta }$$

22 $$v_{r\alpha } = R_{r} i_{r\alpha } + p\psi_{r\alpha } + \omega_{r} \psi_{r\beta }$$

23 $$v_{r\beta } = R_{r} i_{r\beta } + p\psi_{r\beta } - \omega_{r} \psi_{r\alpha }$$

The DTC flux of stator formulas are

24 $$\psi_{s\alpha } = \smallint \left( {v_{s\alpha } - R_{s} i_{s\alpha } } \right)dt$$

25 $$\psi_{s\beta } = \smallint \left( {v_{s\beta } - R_{s} i_{s\beta } } \right)dt$$

26 $$\left| {\psi_{s} } \right| = \sqrt {\psi_{s\alpha }^{2} + \psi_{s\beta }^{2} }$$

27 $$\theta_{f}^{{}} = \arctan \left( {\psi_{s\alpha } /\psi_{s\beta } } \right)$$

The electrical torque of the MSPIM is shown in Eq. (9).

28 $$T_{d} = \frac{3}{2}n_{m} \left( {i_{s\beta } \psi_{s\alpha } - i_{s\alpha } \psi_{s\beta } } \right)$$

The use of formulae (1) through (9) allows for the computation of stator flux and electrical torque. The computation results can then be used to construct closed-loop control for the stator flux and electrical torque. By finding the flux linkage in space and reading the switch table, one can also ascertain the switch state of the control system. Based on the voltage and the inverter’s working state, the flux route flows in the direction of the voltage.

## The proposed FPID controller and SMC of MSPIM

### Sliding mode control design for speed tuning

The choice of sliding mode surface is the first step in the design of a sliding mode controller, which is followed by the design of the sliding mode controller^45^. The controller is made to guarantee the existence or arrival conditions of the sliding mode, allowing the system to reach the sliding mode surface and stay there for a predetermined amount of time^46^. The sliding mode surface is chosen so that the system can function as desired once it reaches it.

The goal of SMC is to drive the speed error to zero and maintain it there. To ensure fast convergence and robustness, the sliding surface can be augmented with an integral term:

To boost the performance of the system, a closed-loop DTC integrated with an SVPWM inverter was included. The difference between the speeds measured and reference (*n*_*m*_ and *n*_*r*_, respectively) is known as the error speed (*er*(t)). The size and sign of the error signal may be determined using (29). The SMC controller modifies the motor reference torque (*T*_d_*) deviation as follows to make up for this error:

29 $$e\left( t \right) = n_{r} - n_{m}$$

30 $$T_{d}^{*} = \alpha_{0} e\left( t \right) + \alpha_{1} \smallint e\left( t \right)dt + \alpha_{2} \dot{e}\left( t \right)$$

Where $\alpha_{0} , \alpha_{1} , \alpha_{2}$ are a is a positive tuning parameter that determines the convergence rate.

**Fig. 4:**
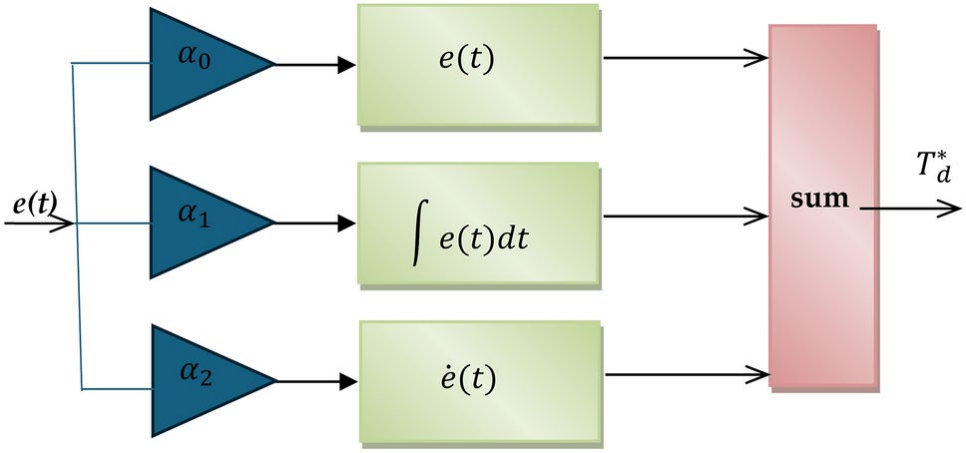
Schematic diagram of SMC.

Figure 4 shows the Simulink block model of the SMC strategy used to calculate the motor reference torque deviation.

The direct reference voltage *v*_d_*

31 $$e1\left( t \right) = \psi_{a}^{*} - \psi_{a}$$

32 $$v_{d}^{*} = K_{p1} e1\left( t \right) + K_{i1} \smallint e1\left( t \right)dt + K_{d1} \frac{de1\left( t \right)}{{dt}}$$

The quadrature reference voltage *v*_q_*

33 $$e2\left( t \right) = T_{d}^{*} - T_{d}$$

34 $$v_{q}^{*} = K_{p2} e2\left( t \right) + K_{i2} \smallint e2\left( t \right)dt + K_{d2} \frac{de2\left( t \right)}{{dt}}$$

The PID factors, K_d1_, K_i1_, K_p1_, K_d2_, K_i2_, and K_p2_ that are utilized to compute the reference voltages *v*_d_* and *v*_q_* are calculated using a PID controller that has been hand-tuned.

The firing angle of controlled switches is obtained using the values of the α- and β-axis voltage components, *v*_*β,*_ and *v*_*α*_, respectively, from Eqs. (39, 40).

35 $$v_{\alpha } = v_{d}^{*} \cos \theta_{f}^{{}} - v_{q}^{*} \sin \theta_{f}^{{}}$$

36 $$v_{\beta } = v_{d}^{*} \sin \theta_{f}^{{}} - v_{q}^{*} \cos \theta_{f}^{{}}$$

### The proposed fuzzy-based PID controller of MSPIM

The schematic recommendation for a closed-loop speed controller for the MSPIM that makes use of fuzzy-based PID control techniques is shown in Fig. 1. The proposed FPID controller aims to maintain the reliability of the MSPIM. The DTC-SVPWM control system is unique because of its simplicity and high speed of MSPIM accuracy^47^. As a consequence, the recommended controller uses the vector control strategy, as depicted in Fig. 1. The fundamental goal is to keep the electromagnetic flux and torque under control.

#### Fuzzy-PID based control strategy with direct torque and SVPWM control

The FPID controller modifies the motor reference trust (*T*_d_*) deviation as follows to make up for this error^39^:

37 $$T_{d}^{*} = K_{p} e\left( t \right) + K_{i} \smallint e\left( t \right)dt + K_{d} \frac{dce\left( t \right)}{{dt}}$$

#### Fuzzy-based PID control system design

To increase the effectiveness of the system, a vector control closed loop was investigated. The discrepancy between the observed and reference speeds is utilized as the error signal. To determine the amount and sign of the error value^48^.

To effectively account for the increasing inaccuracy, the FPID controller generates the changed frequency of the stator part for the MSPIM variance according to the speed deference. The variables *Kd*, *Ki*, and *Kp* that lower the desired function *T*_d_* (t) must be calculated using an FPID controller^49^.

In the proposed FPID controller, the traditional controller is the PID; while the fuzzy controller is treated as the advanced controller that auto-tuned the PID parameters. The fuzzy system receives two signals: the motor speed error and the change of the motor error of speed as input values (*er*(t) and *ce*(t)) and the auto-tuned PID parameters (*Ki*, *Kp*, and Kd) as output functions. The auto-tuned FPID settings are used to regulate the switching signal of the inverter. The *K'p* and *K'd*, as in Eqs. (41) and (42), are between 0 and 1, as shown in^39^ and^47^:

38 $$K^{\prime}_{p} = \left( {K_{p} - K_{pmin} } \right)/\left( {\Delta K_{p} } \right)$$

39 $$K^{\prime}_{d} = \left( {K_{d} - K_{dmin} } \right)/\left( {\Delta K_{d} } \right)$$

Where $\Delta K_{p} = \left( {K_{pmax} - K_{pmin} } \right), \Delta K_{d} = \left( {K_{dmax} - K_{dmin} } \right)$, using the coefficients *βi*, and derivative time constant *Td*, we can get the integral time constant using the following equation:

40 $$T_{i} = \beta_{i} T_{d}$$

**Fig. 5:**
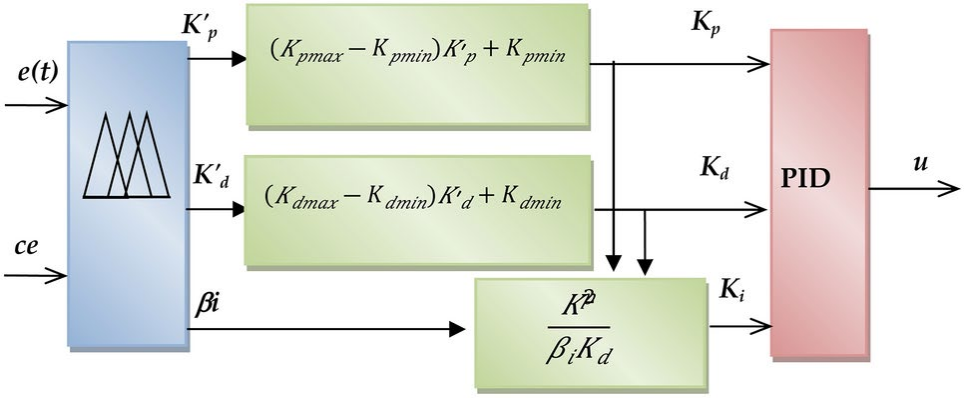
Schematic diagram of Fuzzy PID.

**Fig. 6:**
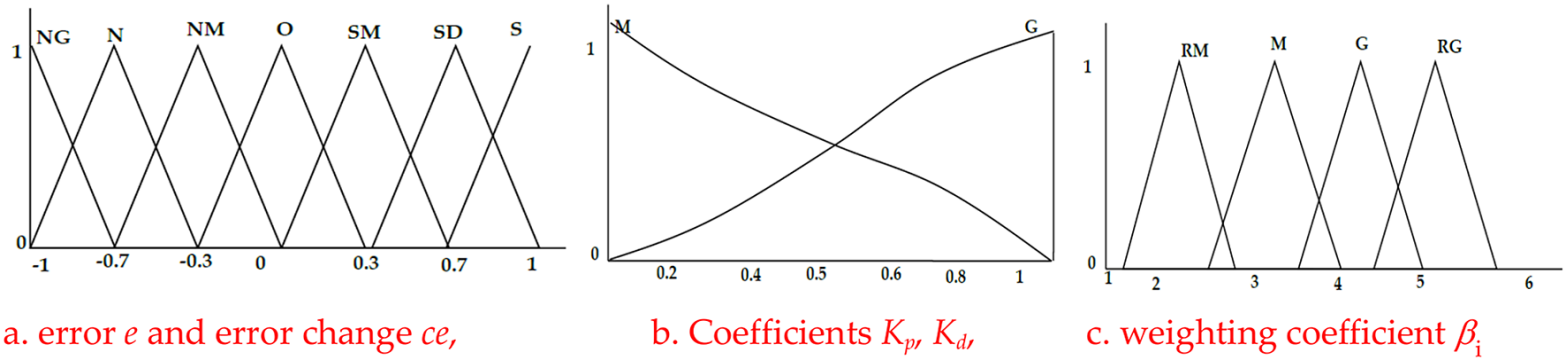
Input and output variable of Fuzzy PID.

Figure 5 illustrates the fuzzy technique for adjusting different PID coefficients to provide a steady control signal. As illustrated in Fig. 6a^50^, the input values are represented in the fuzzy representation by seven overlapping triangular fuzzy memberships. The fuzzification approach is also used for the output variables, as seen in Fig. 6b and c^51^.

The operational range of the two factors (*K*_*p*_ and *K*_*d*_) is calculated, referring to Fig. 6b. Each discourse world is split into two overlapping fuzzy sets. Figure 6.c shows the fuzzy simulation of the coefficient *β*_*i*_ using four fuzzy memberships. Every variable in a fuzzy output or input system has a magnitude of membership ( *μn* in the interval (0,1)) assigned to it. The fuzzy rules listed in Table 1 are used to develop the fuzzy inference system for the variable’s output and input signal.

**Table 1:** The proposed FPID controller truth table of fuzzy rules.

*e*	*ce*	*k*_*p*_	*k*_*d*_	*β*_*i*_	*e*	*ce*	*K*_*p*_	*k*_*d*_	*β*_*i*_
SG, NG	NG	G	M	RM	SM, NM	NG	M	G	G
	ND	G	M	RM		ND	M	G	M
	NM	G	M	RM		NM	G	G	M
	O	G	M	RM		O	G	M	RM
	SM	G	M	RM		SM	G	G	M
	SD	G	M	RM		SD	M	G	M
	SG	G	M	RM		SG	M	G	G
SD, ND	NG	M	G	M	O	NG	M	G	RG
	ND	G	G	M		ND	M	G	G
	NM	G	M	RM		NM	M	G	M
	O	G	M	RM		O	G	G	M
	SM	G	M	RM		SM	M	G	M
	SD	G	G	M		SD	M	G	G
	SG	G	G	M		SG	M	G	RG

where: SG (Positive Big), NG (Negative Big), RG(Large Big), O (Zero), G(Big), NM (Negative Small), ND(Negative Medium), SD(positive Medium), SM(Positive Small), M(Small), RM(Large Small)^51^.

## Applications

### Cases study

**Fig. 7:**
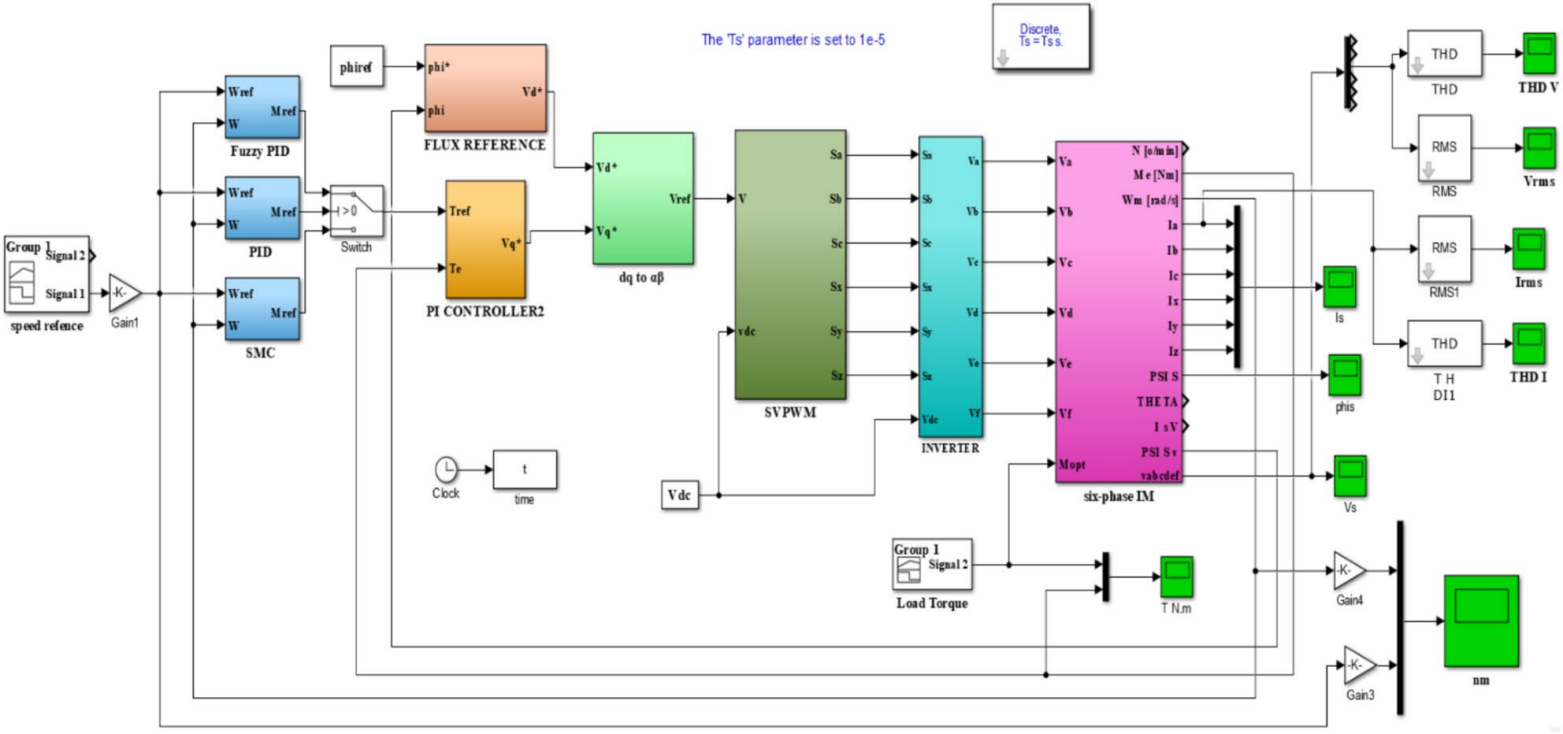
Schematic diagram of DTC MSPIM created in MATLAB/Simulink.

**Fig. 8:**
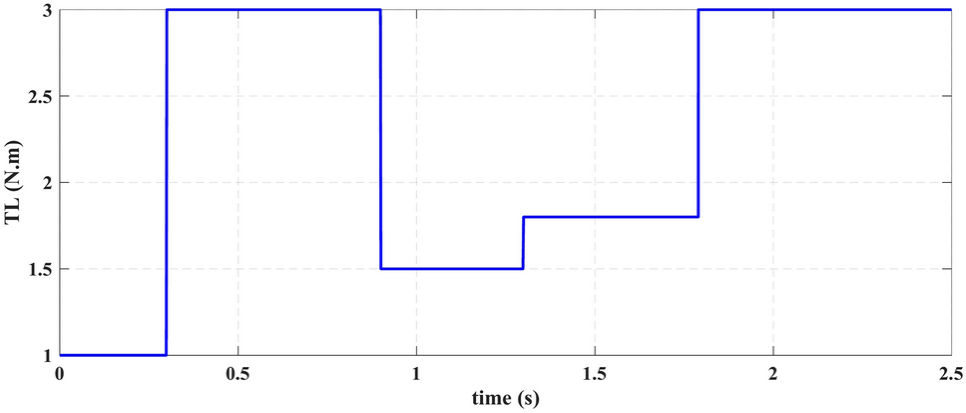
Constant torque-time profile.

Using a modified three-phase IM motor prototype (designed and implemented by rewinding the stator windings to become a six-phase IM motor MSPIM (located in Faculty of Engineering, Kafrelsheikh University) to drive the variable load system and a system model was built using MATLAB/Simulink as shown in Fig. 7. A variable-speed operation with different loading conditions is simulated by the procedure system. The FPID, SMC and PID are also constructed using the DTC technique and integrated with an SVPWM inverter to operate the MSPIM and control torque, speed, and flux. The FPID, SMC, and PID outputs are used as the DTC (DTC-SVM) reference values based on space-vector modulation. The loading behavior under variable speed operation may be defined, as shown in Fig. 8. According to Fig. 8, which depicts the sequential operation of the load variation loaded on MSPIM for the various situations investigated by using PID, SMC and FPID controllers, the sequential operation of the load variation operates as follows:

**Period 1**: In this mode, the MSPIM is operated under a light load (TL = 1 N.m.). In this initial mode, the MSPIM starts at a standstill and builds up gradually to its normal pace. There are 0.1 s left in this time frame.

**Period 2:** (increasing loading): In this mode, 0.5 s after starting, the MSPIM is driven at a higher load (TL = 3 N.m.).

**Period 3:** (Reduced loading): The motor operates in this mode for 0.9 to 1 s at a greater load of (TL = 3 N.m.), after which it lowers the load to (TL = 1.5 N.m.).

**Period 4:** (Reduced loading): During this operational phase, the MSPIM operates at a reduced load of (TL = 1.8 N.m.) for 1.3 to 1.5 s before decreasing the load to (TL = 1.8 N.m.).

**Period 5:** (Reduced loading): The motor operates in this mode for 1.79 s, first at a lower load of TL = 1.8 N.m. and then for 2 s at a higher load of TL = 3 N.m.

**Period 6:** (Full loading): In this operation mode, the MSPIM operates for two to 2.5 s at its rated load (TL = 3 N.m.). Six investigated periods can be grouped to mimic successive operation situations, as shown in Table 2. Table 3 contains the MSPIM parameter data^52^.

**Table 2:** The operation sequence of MSPIM operation.

No	State		Description
	Start	End	
1	0	0.1 s	Starting state
2	0.1 s	0.5 s	Rated speed at 2900 rpm
3	0.5 s	1 s	Lower speed to 2000 rpm
4	1 s	1.5 s	Lower speed to 1500 rpm
5	1.5 s	2 s	Lower speed to 1000 rpm
6	2 s	2.5 s	Return to the rated speed of 2900 rpm

**Table 3:** MSPIM electrical model data.

Symbol	Data	Symbol	Data
*L*_*ls*_ (*H*)	0.0409	*R*_*r*_ (Ω)	8.097
*L*_*m*_ (*H*)	0.849	*J (kg*.*m*^2^)	0.003
*L*_*lr*_ (*H*)	0.0409	*p* (poles)	2
*R*_*s*_ Ω	12	*V*_*ph*_ *(V)*	220
*k*_*p*_	60	*k*_*i*_	2
*k*_*d*_	0.002	*Torque N.m*	3

### Results and discussion

**Fig. 9:**
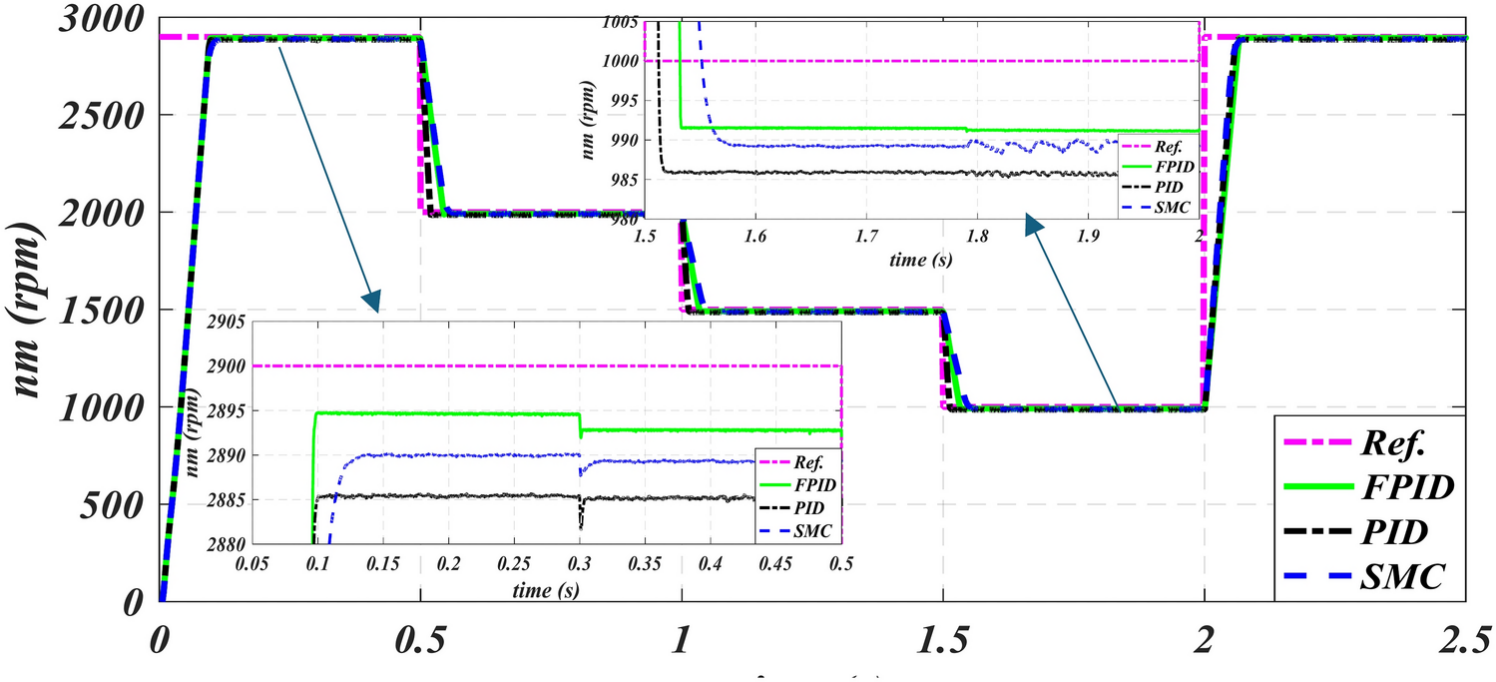
Reference and actual speed time characteristics of PID, SMC and FPID.

**Fig. 10:**
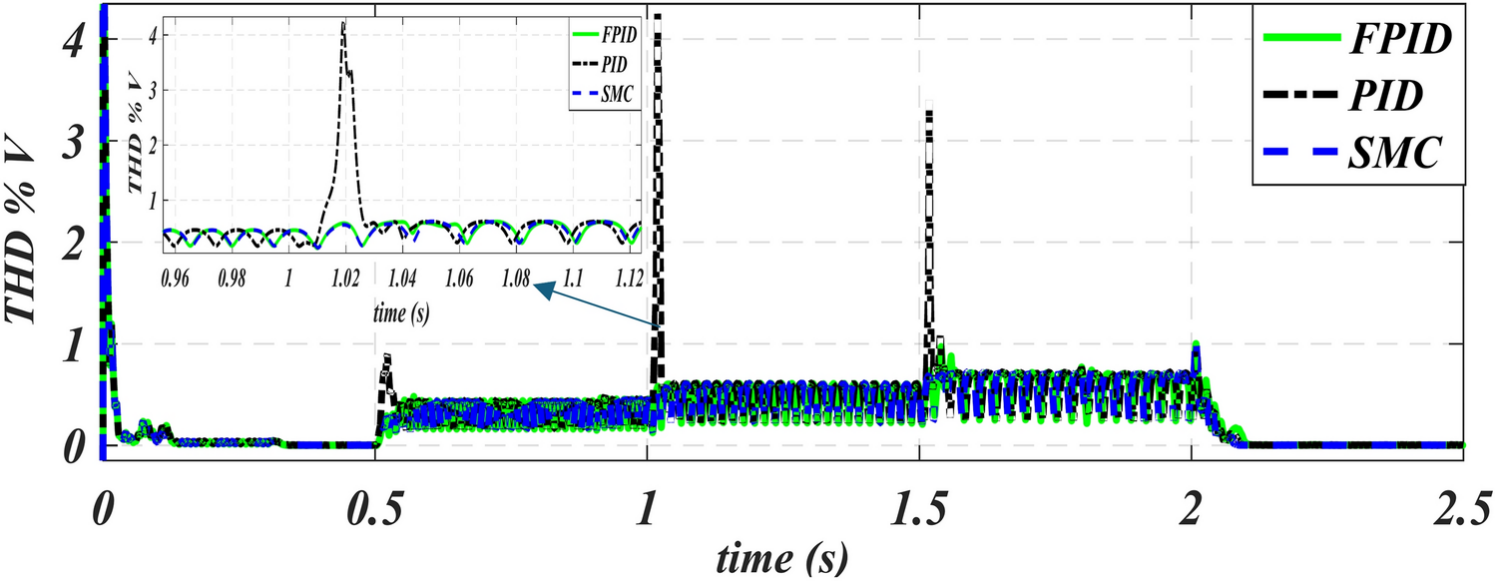
Voltage -time characteristics of THD of PID, SMC and FPID.

**Fig. 11:**
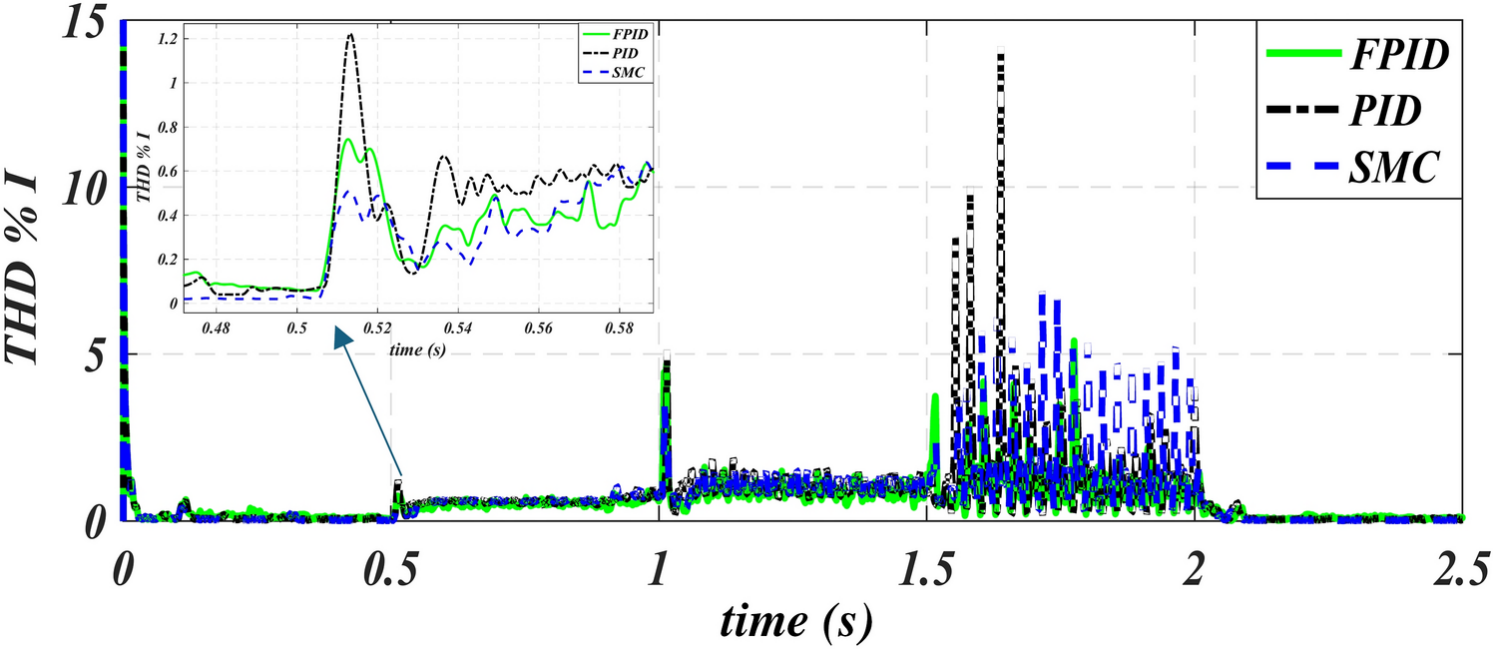
Current–time characteristics of THD of PID, SMC and FPID.

**Fig. 12:**
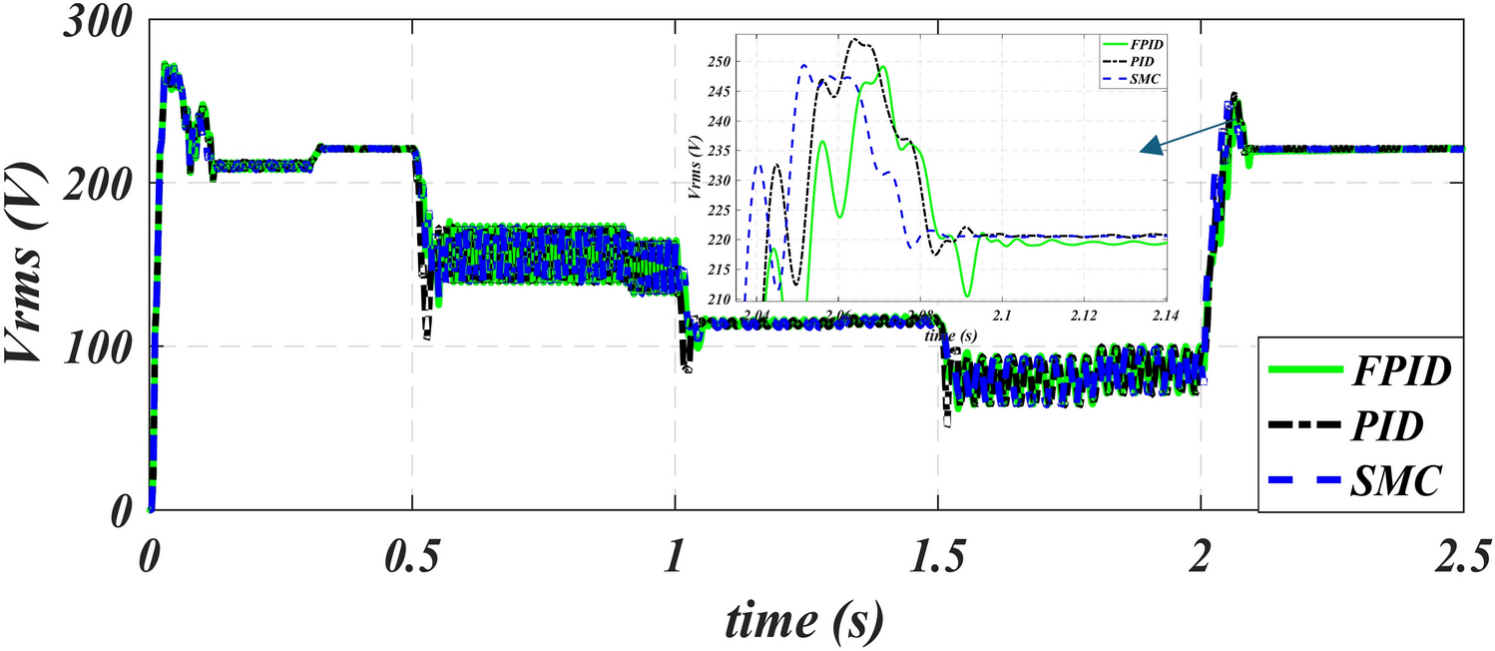
Inverter rms out voltage of PID, SMC and FPID.

**Fig. 13:**
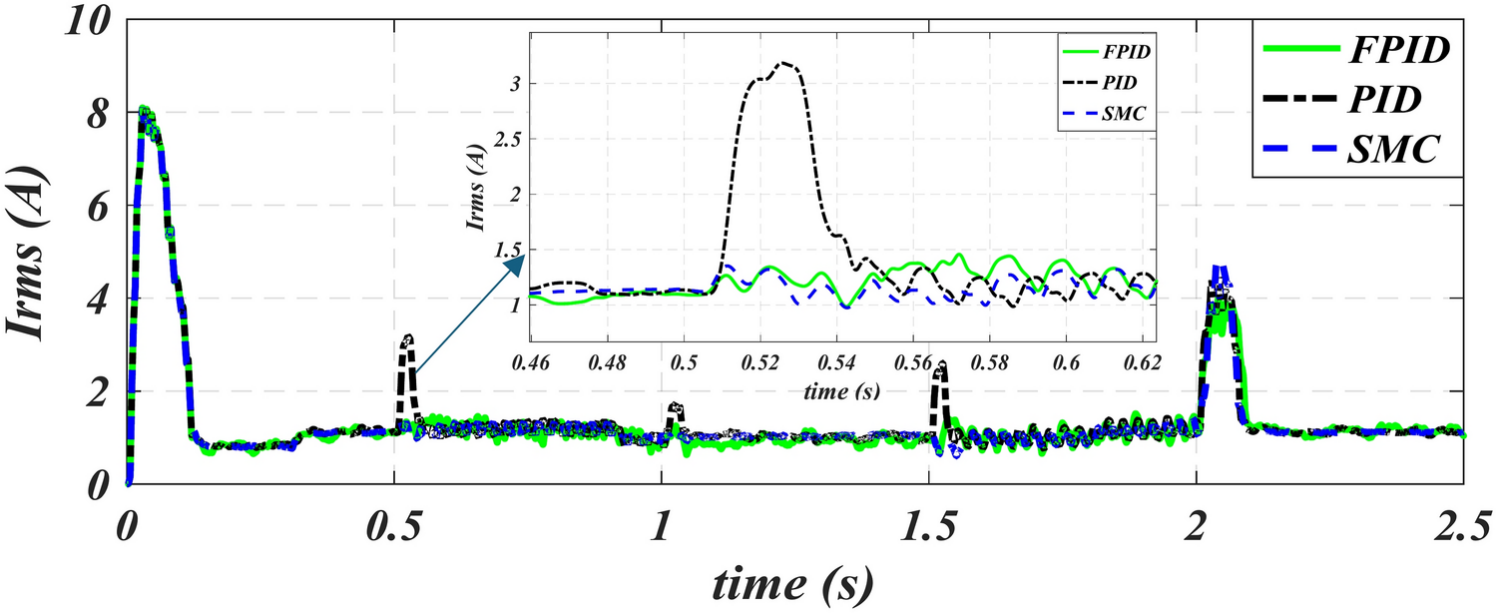
Stator rms current of PID, SMC and FPID.

Figures 9, 10, 11, 12 and 13 display the system under investigation’s simulation results. Figure 9 shows the reference speed variation tracking, enhanced by PID, SMC and FPID controllers, and the modified six-phase induction’s speed tracking under different loading circumstances. In this instance, the FPID controller performs better when there are undershot and steady-state faults. It was found that FPID attains ultimate speed faster than PID and SMC.

Figure 9 illustrates the initial zooming inner graph of the SMC and PID operation, which has a duration of between 0.05 and 0.5 s. This figure shows that, at a loading torque of 3 N.m., the speed error brought on by the fluctuation in load in isn’t larger than 15 rpm in case of PID, 11 rpm in case of SMC and 7 rpm in case of FPID. Figure 9's second inner graph shows zooming, with a length of 1.5 to 2 s. This graph shows that at 1.7 N.m., the speed error brought on by the fluctuation in load in isn’t larger than 14 rpm in case of PID, 11 rpm in case of SMC and 9 rpm in case of FPID.

Figures 10 and 11, respectively, show total harmonic distortion (THD) for the waveforms of voltage and current in three methods. Figure 10 shows that the use of FPID reduces the overshot in THD in voltage special at 1 s from 4.2% to 0.51% and 1.5 s from 3.3% to 0.9%. The zooming figure of THD V at 1.02 s shows that the values ​​obtained using PID are larger than those obtained using the other two methods, MSC and FPID. Figure 11 shows that the use of FPID reduces the overshot in THD in the current special at 1.638 s from 14.1% to 1.9%.

**Fig. 14:**
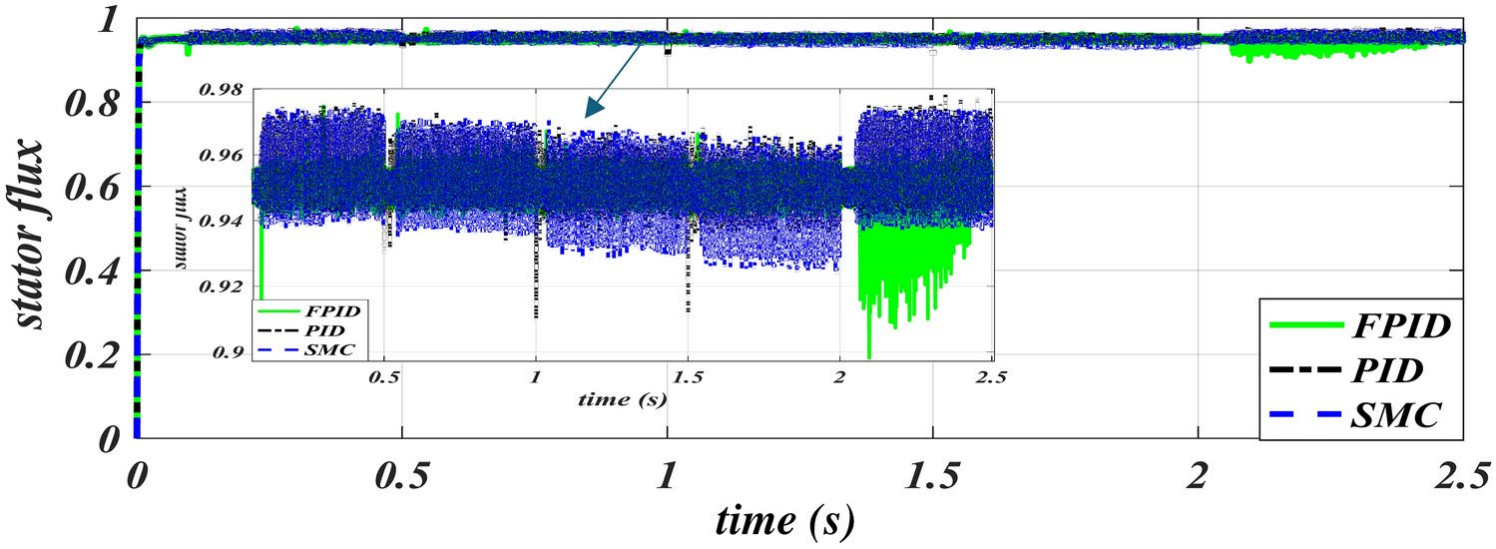
Stator flux-time characteristics of PID, SMC and FPID.

The applied rms voltage of three cases for the six periods under study is shown in Fig. 12. The figure shows that the voltage changes with the change in speed and frequency to fix the flux value within all operating periods to obtain the best performance from the iron core of the machine. As explained in Fig. 13, the stator current in the starting term progressively increases to its greatest value at the beginning instant and falls at a constant speed value of 2900 rpm. The stator’s magnetic flux variation across six periods is shown in Fig. 14. Figure 14 also shows that using FPID reduces the undershot in the stator flux value is fixed at 0.95 web and improves the profile of the stator flux relationship except for the last period, we notice that the volatility of the flux pattern is higher than the other two methods.

Table 4 presents a comparison between the traditional PID (TPID) controller, SMC and the suggested fuzzy-based PID (FPID) controller. It highlights the benefits of FPID controllers over SMC and TPID controllers. The comparison of the three methods used to control a modified six-phase induction motor during operation in periods 2 to 6 can be summarized in the following points:

- The rising time is reduced in second period from 0.12 s in case SMC to 0.1 s in case PID to 0.096 s in proposed FPID. in third period from 0.57 s in case SMC to 0.54 s in case FPID to 0.53 s in PID.
- The greatest level of overall current harmonic distortion in Case 5 decreased from 14.2 in case PID to 6.8 in case SMC to 4.6 in proposed FPID. The greatest level of overall voltage harmonic distortion in Case 5 decreased from 3.4 in case PID to 0.9 in case FPID to 0.7 in SMC. These THDI and THDV values are within the allowable harmonic limit.
- In each of the scenarios that were looked at, the observed speed (OS) with the suggested FPID controller is extremely close to the refractive speed. The steady state speed error (SSE) is reduced by 1.5 to 9.5 rpm for various case studies.
- The stator current overshot is improved by the from 4.52 A in case SMC to 4.48 A in case PID to 4 A in FPID in periods 6.
- Reduce the under-shot of flux from 0.04 in case SMC to 0.03 in case PID to 0.025 in FPID in periods 4.

**Table 4:** Evaluation of SMC, FPID versus TPID controllers.

Index	Controller	Period 2		Period 3		Period 4		Period 5		Period 6	
		OS	SSE	OS	SSE	OS	SSE	OS	SSE	OS	SSE
Speed (rpm)	TPID	4	15–15.5	6	15	1	14	1	14	0.6	14.5
	SMC	3	10–11	1	9	2	11	2	11	0.5	10.5
	FPID	3	5–7	5	7.5	1	8	0.5	9	0.2	5
Current (A)	TPID	8	0.8–1.15	3.4	1.2	1.7	1	2.6	1	4.48	1.1
	SMC	8	0.8–1.1	1.3	1.2	1.18	1	1.4	1	4.52	1.1
	FPID	8	0.8–1.1	1.4	1.2	1.25	0.9	1.5	1	4	1.1
THD V (%)	TPID	1.4	0.1–0.03	0.9	0.3	4.3	0.4	3.4	0.5	1.13	0.03
	SMC	1.4	0.1–0.03	0.42	0.3	0.51	0.4	0.7	0.5	0.95	0.03
	FPID	1.4	0.1–0.03	0.42	0.3	0.51	0.4	0.9	0.5	1	0.03
THD I (%)	TPID	8	0.1–0.09	1.3	0.6	5.1	1	14.2	2	4	0.05
	SMC	8	0.1-0.08	1	0.6	3.6	1	6.8	3.5	1.6	0.04
	FPID	8	0.1–0.15	0.7	0.5	4.7	0.9	4.6	1	1.48	0.9
Flux	TPID	0.03	0.95	0.015	0.95	0.03	0.95	0.03	0.95	0.035	0.95
	SMC	0.04	0.95	0.035	0.95	0.04	0.95	0.04	0.95	0.037	0.95
	FPID	0.06	0.95	0.027	0.95	0.025	0.95	0.025	0.95	0.057	0.95
Rise time (sec.)	TPID	0.1		0.53		1.02		1.52		2.065	
	SMC	0.12		0.57		1.07		1.57		2.09	
	FPID	0.096		0.54		1.035		1.532		2.066	

**Fig. 15:**
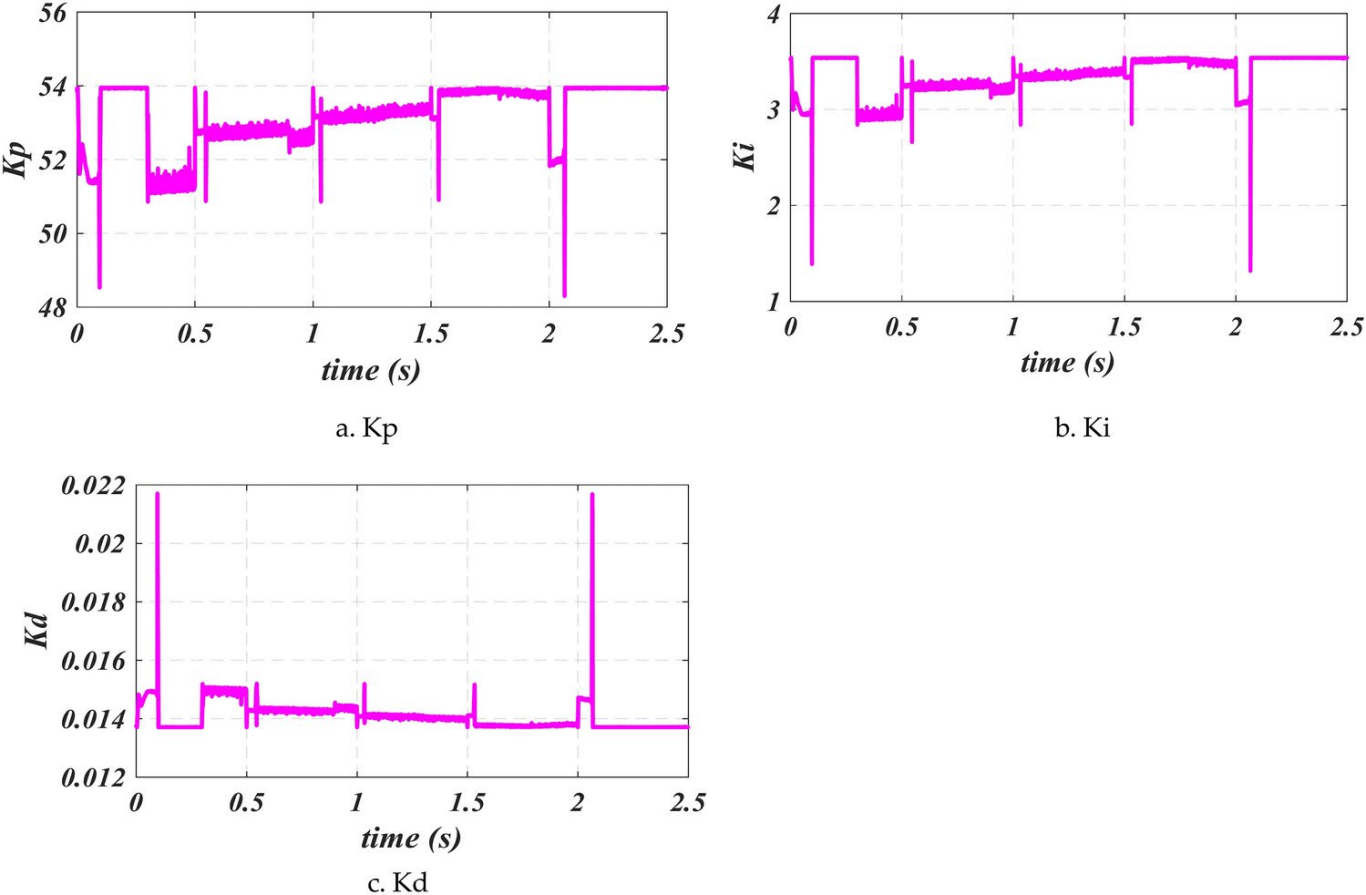
Parameters for Fuzzy auto-tuning FPID control (*Kp*, *Kd*, and *Ki*).

Figure 15 shows that to improve operational performance, *Kp*, *Ki*, and *Kd* are tracked following speed changes. Specifically, *Kp* is tracked between 56 and 48, *Kd* between 0.012 and 0.022, and *Ki* between 1 and 4 per speed change to improve the system performance.

This research was conducted on a prototype manufactured by rewinding a three-phase induction motor to operate as a six-phase induction motor. It can be applied practically to any capacity, so there are no limits to the application of the proposed model under study.

## Conclusions

This study compared the performance of Direct Torque Control (DTC) for a modified six-phase induction motor (MSPIM) driven by a PWM inverter using three control strategies: PID, Sliding Mode Control (SMC), and the proposed Fuzzy PID (FPID) using MATLAB/Simulink. The objective was to enhance motor performance in terms of torque and flux control, dynamic response, and robustness under varying operating conditions.Using a six-phase DTC closed loop applied to MSPIM, the PID, SMC and FPID controllers were developed. It was observed that the THD values of voltage and current were decreased by the PWM inverter, according to the analysis results. If the suggested FPID controller is used, the steady-state error and speed overshot for scenarios 2 through 6 are reduced. With the intentional FPID, the stator undershot current is decreased by 0.9 A in periods 4, in comparison to TPID and SMC controllers. The maximum amount of total harmonic distortion of current in Case 3 is reduced from 1.3 by PIDto 1 by SMC and 0.7 by using FPID. These THDI values are within the allowable harmonic limit. The observed speed using the recommended FPID controller is very close to the refractive speed in all of the examined cases. However, the errors of the conventional estimator range from However, the errors in the traditional range of estimators. The main challenges were the computational complexity and the need for careful tuning of the fuzzy rules and membership functions.

In future work, we plan to compare our suggested system with several methods such as Backstepping, predictive control, etc. To confirm the system’s efficacy, we will perform a series of lab tests on the complete system and contrast it with the computational outcomes. We will use Sensing-less fuzzy direct torque control to set the MRAS method’s six-induction motor speed. We will examine how well the six-phase induction motor performs while using PI and PID in the presence and absence of fuzzy.

## Data Availability

Data analysis in the current study is available from the corresponding author on reasonable request.

## Abbreviations

MSPIM: Modified six-phase induction motor
FPID: Fuzzy PID controller
DTC: Direct torque control
DTC-SVM: DTC with the space vector modulation
PID: Proportional- integral -derivative
*ω*_*r*_: Rotor angular speed (r.p.s)
*T*_*d*_: Electrical torque (N m)
*D*: Viscous friction (N m s)
*J*: Moment of inertia (kg m^2^)
SSE: Steady state error
*n*_*m*_: Motor mechanical speed (r.p.m.)
*v*_*sα*_,* v*_*s*__β_: Stator voltage in *α,*β axis (V)
*R*_*s*_, *R*_*r*_: Stator and rotor resistances (Ω)
*i*_*sα*_,* i*_*s*__β_: Stator currents (A)
*i*_*rα*_,*i*_*r*__β_: Rotor currents (A)
*ψ*_*sα*_,*ψ*_*s*__β_: Stator fluxes (wb)
*ψ*_*rα*_,*ψ*_*r*__β_: Rotor fluxes (wb)
*T*_*L*_: Load torque (N.m)
*p*: No of poles
*L*_*m*_*, L*_*s*_*, L*_*r*_: Mutual, stator, rotor inductances (H)
OS: Overshot
*v*_*d*_,*v*_*q*_: Stator voltage in *dq* axis (V)

## References

[1] Abdelwanis, M. I. & Zaky, A. A. Maximum power point tracking in a perovskite solar pumping system with a six-phase induction motor. *Rev. Roumaine Sci. Techn. Sér. Électrotech. Énergét.* **69**(1), 15–20. 10.59277/RRST-EE.2024.1.3 (2024).

[2] Zemmit, A., Messalti, S. & Harrag, A. A new improved DTC of doubly fed induction machine using GA-based PI controller. *Ain Shams Eng. J.* **9**(4), 1877–1885. 10.1016/j.asej.2016.10.011 (2018).

[3] Shata, A. M. et al. Improved mathematical modeling of six phase induction machines based on fractional calculus. *IEEE Access* **9**, 53146–53155. 10.1109/ACCESS.2021.3069963 (2021).

[4] Albedran, H., Alsamia, S. & Koch, E. Flower fertilization optimization algorithm with application to adaptive controllers. *Sci. Rep.* **15**(1), 6273. 10.1038/s41598-025-89840-1 (2025). 39979357 10.1038/s41598-025-89840-1 PMC11842593

[5] Guo, K., Zhang, H., Wei, C., Jiang, H. & Wang, J. Novel sliding mode control of the manipulator based on a nonlinear disturbance observer. *Sci. Rep.* **14**(1), 30656. 10.1038/s41598-024-77125-y (2024). 39730400 10.1038/s41598-024-77125-y PMC11680702

[6] Mohamed, M. A. E., Jagatheesan, K. & Anand, B. Modern PID/FOPID controllers for frequency regulation of interconnected power system by considering different cost functions. *Sci. Rep.* **13**(1), 14084. 10.1038/s41598-023-41024-5 (2023). 37640919 10.1038/s41598-023-41024-5 PMC10462752

[7] Abdelwanis, M. I. & Ragab, E. L. Efficient parameter estimation procedure using sunflower optimization algorithm for six-phase induction motor. *Revue Roumaine Sci. Tech. Sér. Électrotech. Énergét.* **67**(3), 259–264 (2022).

[8] Sun, J., Zheng, Z., Li, C., Wang, K. & Li, Y. Optimal fault-tolerant control of multiphase drives under open-phase/open-switch faults based on DC current injection. *IEEE Trans. Power Electron.* **37**(5), 5928–5936. 10.1109/TPEL.2021.3135280 (2022).

[9] Antunes, H. R. P., Fonseca, D. S. B. & Cardoso, A. J. M. Modeling of symmetrical six-phase induction machines under stator faults. *IEEE Trans. Ind. Appl.* **59**(3), 3232–3242. 10.1109/TIA.2023.3242940 (2023).

[10] Nabi, H. P., Dadashi, P. & Shoulaie, A. A novel structure for vector control of a symmetrical six-phase induction machine with three current sensors. In *2011 10th International Conference on Environment and Electrical Engineering, EEEIC.EU 2011—Conference Proceedings* 1–5 (2011). 10.1109/EEEIC.2011.5874735.

[11] Sumit-Mandal, M. Performance analysis of six-phase induction motor. *Int. J. Eng. Res. Technol. (IJERT)* **4**(2), 589–593 (2015).

[12] Holakooie, M. H., Ojaghi, M. & Taheri, A. Direct torque control of six-phase induction motor with a novel MRAS-based stator resistance estimator. *IEEE Trans. Ind. Electron.* **65**(10), 7685–7696. 10.1109/TIE.2018.2807410 (2018).

[13] Liu, Z., Li, Y. & Zheng, Z. A review of drive techniques for multiphase machines. *CES Trans. Electr. Mach. Syst.* **2**(2), 243–251. 10.30941/CESTEMS.2018.00030 (2018).

[14] Sola, T. E., Chiu, H. J., Liu, Y.-C. & Rahman, A. N. Improved direct torque control of induction motor for torque ripple minimization. *IEEE Access* **10**, 131980–131995. 10.1109/ACCESS.2022.3230139 (2022).

[15] Sami, I., Ullah, S., Basit, A., Ullah, N. & Ro, J.-S. Integral super twisting sliding mode based sensorless predictive torque control of induction motor. *IEEE Access* **8**, 186740–186755. 10.1109/ACCESS.2020.3028845 (2020).

[16] Zemmit, A. New direct torque control of dual star induction motor using Grey Wolf Optimization Technique. *PRZEGLĄD Elektrotechn.* **1**(2), 111–115. 10.15199/48.2024.02.21 (2024).

[17] Abderrahim, A. et al. New modified direct torque control-fuzzy logic controller of doubly fed induction machine. *Int. J. Adv. Appl. Sci.* **4**(7), 16–20. 10.21833/ijaas.2017.07.004 (2017).

[18] El Ouanjli, N. et al. A new intelligent adaptation mechanism of MRAS based on a genetic algorithm applied to speed sensorless direct torque control for induction motor. *Int. J. Dyn. Control* **10**(6), 2095–2110. 10.1007/s40435-022-00947-z (2022).

[19] Gdaim, S., Mtibaa, A. & Mimouni, M. F. Artificial neural network-based DTC of an induction machine with experimental implementation on FPGA. *Eng. Appl. Artif. Intell.* **121**, 105972. 10.1016/j.engappai.2023.105972 (2023).

[20] El Ouanjli, N., Mahfoud, S., Derouich, A., El Daoudi, S. & El Mahfoud, M. Speed sensorless fuzzy direct torque control of induction motor based MRAS method. *Digital Technol. Appl. ICDTA* **2022**, 779–790. 10.1007/978-3-031-02447-4_80 (2022).

[21] Mahfoud, M., Bossoufi, B., Ouanjli, N., Said, M. & Taoussi, M. Improved direct torque control of doubly fed induction motor using space vector modulation. *Int. J. Intell. Eng. Syst.* **14**(3), 177–188. 10.22266/ijies2021.0630.16 (2021).

[22] Al-Hitmi, M. A., Moinoddin, S., Iqbal, A., Rahman, K. & Meraj, M. Space vector vs. sinusoidal carrier-based pulse width modulation for a seven-phase voltage source inverter. *CPSS Trans. Power Electron. Appl.* **4**(3), 230–243. 10.24295/CPSSTPEA.2019.00022 (2019).

[23] Tenconi, A., Rubino, S. & Bojoi, R. Model predictive control for multiphase motor drives—a technology status review. In *2018 International Power Electronics Conference (IPEC-Niigata 2018 -ECCE Asia)**, **IEEE* 732–739 (2018). 10.23919/IPEC.2018.8507960.

[24] Suhel, S. M. & Maurya, R. Modelling, design and analysis of multi-phase induction motor. *Int. J. Power Energy Convers.* **8**(2), 186. 10.1504/IJPEC.2017.083195 (2017).

[25] Ochoa, D., Martinez, S. & Arévalo, P. A novel fuzzy-logic-based control strategy for power smoothing in high-wind penetrated power systems and its validation in a microgrid lab. *Electron. (Basel)* **12**(7), 1721. 10.3390/electronics12071721 (2023).

[26] Belay, A., Salau, A. O., Kassahun, H. E. & Eneh, J. N. Stator flux estimation and hybrid sliding mode torque control of an induction motor. *Int. J. Syst. Assuranc. Eng. Manage.*10.1007/s13198-024-02275-1 (2024).

[27] Kali, Y., Rodas, J., Doval-Gandoy, J., Ayala, M. & Gonzalez, O. Enhanced reaching-law-based discrete-time terminal sliding mode current control of a six-phase induction motor. *Machines* **11**(1), 107. 10.3390/machines11010107 (2023).

[28] Maghfiroh, H., Saputro, J. S., Adriyanto, F., Sujono, A. & Lambang, R. L. Performance evaluation of fuzzy-PID in speed control of three phase induction motor. *IOP Conf. Ser. Mater. Sci. Eng.* **1096**(1), 012071. 10.1088/1757-899X/1096/1/012071 (2021).

[29] Liu, Q. et al. Research on speed tracking of asynchronous motor based on fuzzy control and vector control. In *2020 39th Chinese Control Conference (CCC)**, **IEEE* 2144–2149 (2020). 10.23919/CCC50068.2020.9188846.

[30] Khanh, P. Q. & Anh, H. P. H. Advanced PMSM speed control using fuzzy PI method for hybrid power control technique. *Ain Shams Eng. J.* **2023**, 102222. 10.1016/j.asej.2023.102222 (2023).

[31] Mahfouz, M., Taiomour, A., Ashry, M. M. & Elnashar, G. PID vs backstepping control for cooperative quadrotors unmanned aerial vehicles. *IOP Conf. Ser. Mater. Sci. Eng.* **610**(1), 012057. 10.1088/1757-899X/610/1/012057 (2019).

[32] Boukhalfa, G., Belkacem, S., Chikhi, A. & Benaggoune, S. Genetic algorithm and particle swarm optimization tuned fuzzy PID controller on direct torque control of dual star induction motor. *J. Cent. South Univ.* **26**(7), 1886–1896. 10.1007/s11771-019-4142-3 (2019).

[33] Luo, M. et al. Full-order adaptive sliding mode control with extended state observer for high-speed PMSM speed regulation. *Sci. Rep.* **13**(1), 6200. 10.1038/s41598-023-33455-x (2023). 37069198 10.1038/s41598-023-33455-x PMC10110560

[34] Zahraoui, Y., Moutchou, M., Tayane, S., Fahassa, C. & Elbadaoui, S. Induction motor performance improvement using super twisting SMC and twelve sector DTC. *Int. J. Robot. Control Syst.* **4**(1), 50–68. 10.31763/ijrcs.v4i1.1090 (2024).

[35] El-Daoudi, S., Lazrak, L., El-Ouanjli, N. & Ait-Lafkih, M. Applying sliding mode technique for the nonlinear DTC-SPWM control strategy of sensorless squirrel cage asynchronous motor. *Int. J. Dyn. Control* **9**(4), 1633–1644. 10.1007/s40435-021-00758-8 (2021).

[36] Salem, A. & Narimani, M. A review on multiphase drives for automotive traction applications. *IEEE Trans. Transport. Electr.* **5**(4), 1329–1348. 10.1109/TTE.2019.2956355 (2019).

[37] Shawier, A., Habib, A., Mamdouh, M., Abdel-Khalik, A. S. & Ahmed, K. H. Assessment of predictive current control of six-phase induction motor with different winding configurations. *IEEE Access* **9**, 81125–81138. 10.1109/ACCESS.2021.3085083 (2021).

[38] Hammad, R., Dabour, S. M. & Rashad, E. M. Asymmetrical six-phase induction motor drives based on Z-source inverters: Modulation, design, fault detection and tolerance. *Alex. Eng. J.* **61**(12), 10055–10070. 10.1016/j.aej.2022.02.058 (2022).

[39] Abdelwanis, M. I. & El-Sehiemy, R. A. A fuzzy-based controller of a modified six-phase induction motor driving a pumping system. *Iran. J. Sci. Technol. Trans. Electr. Eng.*10.1007/s40998-018-0066-4 (2019).

[40] Chinmaya,K. A. & Singh, G. K. Analysis of space vector PWM techniques for dual three-phase induction machine. In *2017 Innovations in Power and Advanced Computing Technologies (i-PACT), IEEE* 1–5 (2017). 10.1109/IPACT.2017.8245131.

[41] Lim, C. S., Lee, S. S. & Levi, E. Continuous-control-set model predictive current control of asymmetrical six- phase drives considering system nonidealities. *IEEE Trans. Industr. Electron.* **70**(8), 7615–7626. 10.1109/TIE.2022.3206703 (2023).

[42] Salau, A. O. & Anteneh, T. M. Direct quadrate modeling of a direct torque control for a 3-phase induction motor. In *2021 6th International Conference on Signal Processing, Computing and Control (ISPCC)**, **IEEE* 522–527 (2021). 10.1109/ISPCC53510.2021.9609480.

[43] Anteneh, E. A., Salau, T. M., Agajie, A. O. & Hailu, T. F. Design and implementation of a direct torque controller for a three phase induction motor based on DSP. *Int. J. Appl. Eng. Res.* **14**(22), 4181–4187 (2019).

[44] Abdelwanis, M. I., Rashad, E. M., Taha, I. B. M. & Selim, F. F. Implementation and control of six-phase induction motor driven by a three-phase supply. *Energies* **14**(22), 1–16 (2021).

[45] Zhu, Q. Complete model-free sliding mode control (CMFSMC). *Sci. Rep.* **11**(1), 22565. 10.1038/s41598-021-01871-6 (2021). 34799614 10.1038/s41598-021-01871-6 PMC8604951

[46] Barth, R. et al. Testing pseudotopological and nontopological models for SMC-driven DNA loop extrusion against roadblock-traversal experiments. *Sci. Rep.* **13**(1), 8100. 10.1038/s41598-023-35359-2 (2023). 37208374 10.1038/s41598-023-35359-2 PMC10199080

[47] Abdelwanis, M. I. & El-Sehiemy, R. A. Performance enhancement of split-phase induction motor by using fuzzy-based PID controller. *J. Electr. Eng.* **70**, 103–112. 10.2478/jee-2019-0016 (2019).

[48] Yin, H., Wang, Z., Liu, J. & Liu, P. Steer-by-wire control algorithm using a dual-layer closed-loop model. *Sci. Rep.* **14**(1), 28536. 10.1038/s41598-024-79703-6 (2024). 39557948 10.1038/s41598-024-79703-6 PMC11574273

[49] Qing, F. Y. et al. A novel fuzzy PID velocity control of linear elevator driven by the permanent magnet linear synchronous motor. In *2008 International Conference on Electrical Machines and Systems, Wuhan, China* 1539–1542 (2008).

[50] Barakat, M. Optimal design of fuzzy-PID controller for automatic generation control of multi-source interconnected power system. *Neural Comput. Appl.* **34**(21), 18859–18880. 10.1007/s00521-022-07470-4 (2022).

[51] Abdelwanis, M. I., El-Sousy, F. F. M. & Ali, M. M. A fuzzy-based proportional–integral–derivative with space-vector control and direct thrust control for a linear induction motor. *Electron. (Basel)* **12**(24), 4955. 10.3390/electronics12244955 (2023).

[52] Rizk-Allah, R. M., Abdelwanis, M. I., El-Sehiemy, R. A. & Abd-Elrazek, A. S. An interior search algorithm based on chaotic and crossover strategies for parameter extraction of polyphase induction machines. *Neural Comput. Appl.*10.1007/s00521-022-08055-x (2022).

